# Death after the Administration of COVID-19 Vaccines Approved by EMA: Has a Causal Relationship Been Demonstrated?

**DOI:** 10.3390/vaccines10020308

**Published:** 2022-02-16

**Authors:** Aniello Maiese, Arianna Baronti, Alice Chiara Manetti, Marco Di Paolo, Emanuela Turillazzi, Paola Frati, Vittorio Fineschi

**Affiliations:** 1 Department of Surgical, Medical and Molecular Pathology and Critical Care Medicine, Institute of Legal Medicine, University of Pisa, Via Roma 55, 56126 Pisa, Italy; aniello.maiese@unipi.it (A.M.); arianna-baronti@virgilio.it (A.B.); a.manetti3@studenti.unipi.it (A.C.M.); marco.dipaolo@unipi.it (M.D.P.); emanuela.turillazzi@unipi.it (E.T.); 2 Department of Anatomical, Histological, Forensic and Orthopedic Sciences, Institute of Legal Medicine, Sapienza University of Rome, Viale Regina Elena 336, 00161 Rome, Italy; paola.frati@uniroma1.it

**Keywords:** COVID-19, vaccine, death, side effects

## Abstract

More than eight billion doses of COVID-19 vaccines have been administered globally so far and 44.29% of people are fully vaccinated. Pre-authorization clinical trials were carried out and the safety of vaccines is still continuously monitored through post-commercialization surveillance. However, some people are afraid of vaccine side effects, claiming they could lead to death, and hesitate to get vaccinated. Herein, a literature review of COVID-19-vaccine-related deaths has been carried out according to the PRISMA standards to understand if there is a causal relationship between vaccination and death and to highlight the real extent of such events. There have been 55 cases of death after COVID-19 vaccination reported and a causal relationship has been excluded in 17 cases. In the remaining cases, the causal link between the vaccine and the death was not specified (8) or considered possible (15), probable (1), or very probable/demonstrated (14). The causes of deaths among these cases were: vaccine-induced immune thrombotic thrombocytopenia (VITT) (32), myocarditis (3), ADEM (1), myocardial infarction (1), and rhabdomyolysis (1). In such cases, the demonstration of a causal relationship is not obvious, and more studies, especially with post-mortem investigations, are needed to deepen understanding of the possible pathophysiological mechanisms of fatal vaccine side effects. In any event, given the scarcity of fatal cases, the benefits of vaccination outweigh the risks and the scientific community needs to be cohesive in asserting that vaccination is fundamental to containing the spread of SARS-CoV-2.

## 1. Introduction

Since severe acute respiratory syndrome coronavirus 2 (SARS-CoV-2) has spread worldwide, the international scientific community has been focused on developing strategies to contain it [1,2]. Thanks to its efforts, to date, SARS-CoV-2 infection can be counteracted not only with simple preventative measures (e.g., face masks, handwashing, and physical distancing) but also with effective vaccines [3,4]. According to the Centers for Disease Control and Prevention (CDC), a vaccine is “a preparation that is used to stimulate the body’s immune response against diseases” [5]. More than eight billion doses of COVID-19 vaccines have been administered globally so far, and 44.29% of people are fully vaccinated [6,7]. Regarding the European Union, about 660 million doses have been administered and more than 290 million (298,845,193) people are fully vaccinated [8]. COVID-19 vaccines, due to the urgency of the global pandemic, were given emergency approval [9,10,11,12]. Clinical trials counting thousands of participants were carried out before the authorization [13,14]. Moreover, since the vaccines’ administration to the population was authorized, their safety has been continuously monitored [15,16]. However, some people are afraid of vaccines’ side effects, claiming they could lead to death, and hesitate to get vaccinated. Herein, we report a literature review concerning cases of death following these vaccines’ administration to understand if there is a causal relationship and to highlight the real extent of such events.

## 2. Materials and Methods

The present systematic review was carried out according to the Preferred Reporting Items for Systematic Review (PRISMA) standards [17]. A systematic literature search and a critical review of the collected studies were conducted. An electronic search of PubMed, Science Direct Scopus, Google Scholar, and Excerpta Medica Database (EMBASE) from database inception to November 2021 was performed. The search terms were “COVID-19”, “SARS-CoV-2”, “vaccine”, “vaccination”, “death”, and “autopsy” in the title, abstract, and keywords. 

The bibliographies of all located papers were examined and cross-referenced to further identify relevant literature. A methodological appraisal of each study was conducted according to the PRISMA standards, including an evaluation of bias. The data collection process included study selection and data extraction. Two researchers (AB and ACM) independently examined the papers with titles or abstracts that appeared to be relevant and selected those that concerned cases of death after COVID-19 vaccination. Papers regarding anaphylaxis deaths were excluded, as were preprints and non-English articles. Disagreements concerning eligibility among the researchers were resolved by consensus. Data extraction was performed by two investigators (AB and ACM) and verified by two other investigators (AM and MDP). Two investigators drafted the manuscript (AB and ACM) and other investigators (ET, PF, and VF) revised and finalized it. This study was exempt from institutional review board approval, as it did not involve human subjects.

## 3. Results

**Figure 1:**
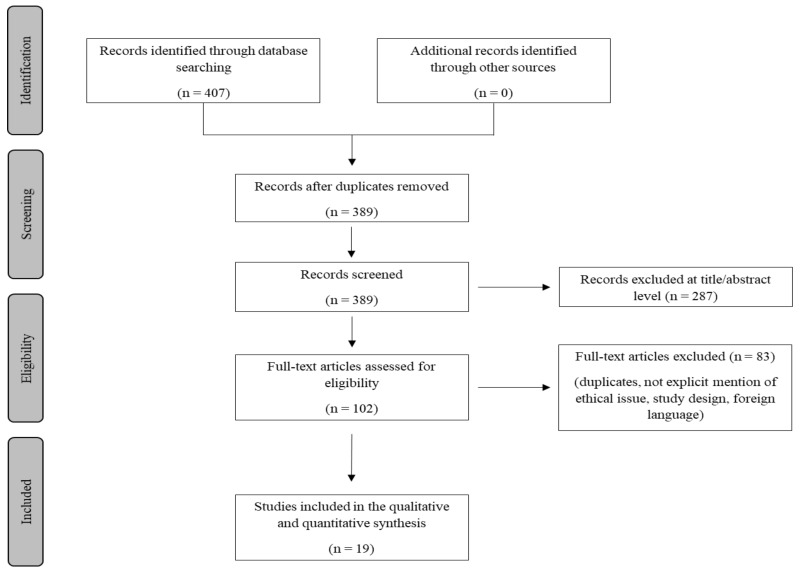
Methodology search strategy: we identified 389 articles after removing duplicates, the screening based on their abstracts left 102 studies, and after a careful evaluation based on the aims of this review 19 research articles were included.

A review of the titles and abstracts as well as a manual search of the reference lists were carried out. The reference lists of all identified articles were reviewed to find missed literature. This search identified 407 articles, which were then screened to exclude duplicates. The resulting 389 reference lists were screened based on their abstract, which left 102 articles for further consideration. In addition, non-English papers were excluded, and the following inclusion criteria were used: (1) original research articles, (2) reviews and mini-reviews, and (3) case reports/series. These publications were carefully evaluated, taking into account the main aims of the review. Figure 1 illustrates our search strategy. This evaluation left 19 scientific papers comprising original research articles, case reports, and case series.

The 29 articles were then carefully evaluated to extract the useful information. Table 1 shows the results of our research and data extraction.

The sex and age of the subjects was not specified in all papers, therefore we used only papers in which these data were specified for the following calculations. The male/female ratio was close to one (1.04). The mean age was 52.74 years (range 22–91). The cases of death of people aged 50 years or younger were 21, while the cases of death of people older than 50 years were 26, with a ratio of 0.8. Considering the types of COVID-19 vaccines authorized by EMA (BNT162b2, Comirnaty^®^, BioNTech/Pfizer; mRNA1273, Spikevax^®^, Moderna; adenovirus type 26 vector COVID-19 Vaccine Janssen, Janssen^®^, Johnson&Johnson; recombinant chimpanzee adenoviral vector vaccine ChAdOx1 nCoV-19, Vaxzevria^®^, AstraZeneca), we found a total of 55 cases of death reported in the literature. 

**Figure 2:**
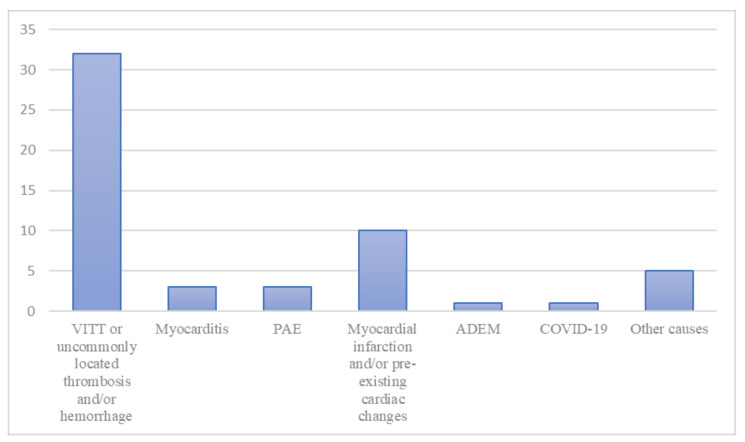
The distribution of the cause of death among the cases. “Other causes” includes one case of massive cerebral hemorrhage not associated with thrombosis or auto-antibodies, one case of anaphylactic reaction to anesthetics associated with cerebral venous sinus thrombosis and anti-PF4 antibodies, one case of hyperglycemic coma, one case of hemorrhagic shock due to aortic dissection and rupture, and one case of death due to the complications of rhabdomyolysis. ADEM indicates acute disseminated encephalomyelitis; COVID-19, coronavirus disease 2019; PAE, pulmonary embolism; VITT, vaccine-induced immune thrombotic thrombocytopenia.

The causes of death were: vaccine-induced immune thrombotic thrombocytopenia (VITT) or, when VITT was not completely investigated, uncommonly located thrombosis associated with hemorrhages, in 32 cases (58.1%); myocardial infarction and/or some kind of pre-existing cardiac changes in ten cases (18.2%); myocarditis in three cases (5.4%); pulmonary artery embolism (PAE) in three cases (5.4%); acute disseminated encephalomyelitis (ADEM) in one case (1.8%); massive cerebral hemorrhage not associated with thrombosis or auto-antibodies in one case (1.8%); anaphylactic reaction to anesthetics associated with cerebral venous sinus thrombosis and anti-PF4 antibodies in one case (1.8%); hyperglycemic coma in one case (1.8%); hemorrhagic shock due to aortic dissection with rupture in one case (1.8%); complications of rhabdomyolysis in one case (1.8%). Figure 2 shows the distribution of the causes of death among the cases.

**Figure 3:**
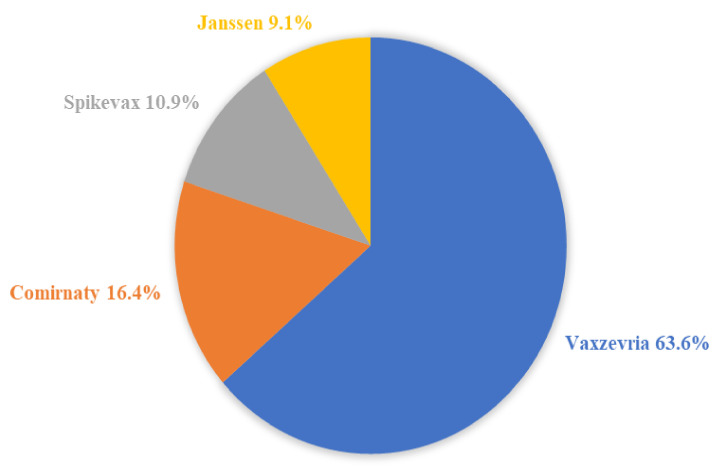
The distribution of the type of vaccine among the 55 cases of death after the vaccination.

The distribution concerning the type of vaccine was as follows: 35 cases of death following the Vaxzevria^®^ (63.6%), 9 cases following the Comirnaty^®^ (16.4%), 6 cases following the Spikevax^®^ (10.9%), and 5 cases following the Janssen^®^ (9.1%). Figure 3 illustrates the distribution of the type of vaccine among the 55 cases.

**Figure 4:**
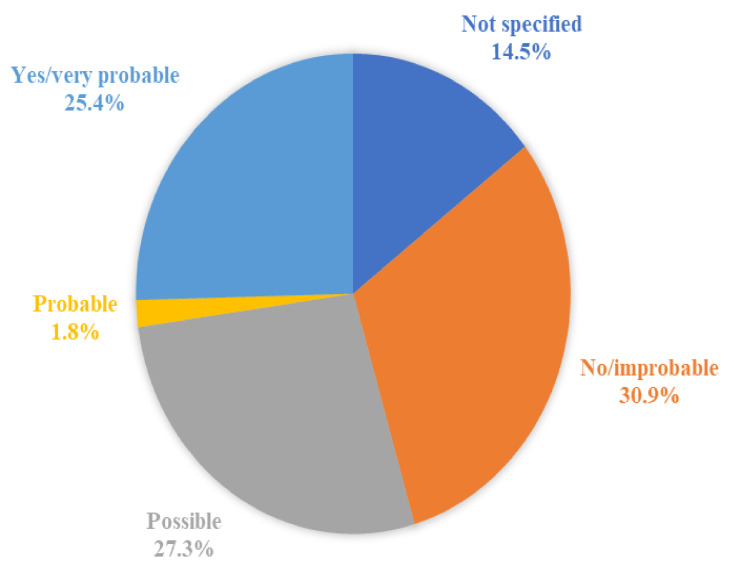
The distribution of the probability of the causal relationship among the 55 cases of death after the vaccination.

The causal relationship between the death and the vaccine was not specified (meaning that the authors did not explicitly state if there was a causal relation or not) in eight cases (14.5%), not demonstrated or improbable in 17 cases (30.9%), possible in 15 cases (27.3%), probable in one case (1.8%), and demonstrated or very probable in 14 cases (25.4%). Figure 4 shows the distribution of the probability of the causal relationship among the 55 cases.

In Table 2, the distribution of causes of death per type of vaccine is shown. The cases in which the causal relationship between the vaccine and the death was not demonstrated or was improbable have not been included in this table.

## 4. Discussion

According to WHO, there have been more than 260 million cases of COVID-19 infection and more than 5 million deaths reported globally, underlining the importance of an effective vaccination program as a powerful weapon to counteract the virus [6]. At the time of writing this paper, the European Medical Agency (EMA) has approved four vaccines: BNT162b2 (Comirnaty^®^, BioNTech/Pfizer, New York, NY, USA), mRNA1273 (Spikevax^®^, Moderna, Cambridge, MA, USA), Ad26.COV2.S (Janssen^®^, Johnson&Johnson, New Brunswick, NJ, USA), and ChAdOx1 nCoV-19/AZD1222 (Vaxzevria^®^, AstraZeneca, Cambridge, UK).

Comirnaty^®^ and Spikevax^®^ work by introducing a piece of mRNA that contains the instructions for synthesizing the Spike protein, a surface protein that acts as a key allowing the virus to enter the cells. Once the human cells produce the Spike protein, they rapidly break down the mRNA [37,38,39]. This is the first time an mRNA vaccine has been administered to the population.

Ad26.COV2.S and ChAdOx1 are viral vector vaccines. They use an adenovirus to deliver a DNA genetic sequence encoding for the Spike protein into the cells [40]. Adenoviruses have already been used as vaccine agents in other infectious diseases, such as the Ebola infection [41].

It is necessary to overcome COVID-19 vaccine hesitancy to achieve a high percentage of vaccinated people [42]. However, some people are procrastinating and rejecting the vaccine because of the fear of death due to its side effects. They claim that these vaccines are new and therefore have not been studied enough to be safe [43,44,45,46,47,48,49]. Indeed, COVID-19 vaccines may cause side effects, as any other drug. The most common are mild and short-lived, such as fever, administration-site pain, weakness, etc. [50,51]. In addition, some severe adverse reactions have occurred. Regarding the mRNA vaccines, myocarditis, pericarditis, and anaphylaxis have been described, but these cases are very rare [52,53]. Moreover, it has been demonstrated that the viral vector vaccines can cause venous thrombosis associated with thrombocytopenia, so-called vaccine-induced immune thrombotic thrombocytopenia (VITT), and very few cases of Guillain–Barré syndrome have been reported [54,55,56].

We found 55 cases of death temporally related to COVID-19 vaccine administration. The male/female ratio was close to one (1.04), showing no difference between sexes. The ratio between people aged 50 years or younger and older than 50 years was 0.8. So, it seems there is a slight predominance of older people among the cases. This is quite interesting, as we would have expected a more pronounced difference. Some possible reasons are that the death of a young person arouses greater interest, inducing the performance of investigations, and is more likely to be reported.

As mentioned before, more than eight billion doses of COVID-19 vaccines have been administered worldwide. However, searching the literature, we found only 55 cases of death temporally correlated with vaccination, and in 17 of these a causal relationship has been excluded. Therefore, these 17 cases of death are only temporally, and not causally, related to the vaccine administrations. Among the remaining 38 cases, in eight the probability of a causal correlation between the vaccine and the death was not specified, in 16 the authors stated that it was possible or probable, while in 14 cases the causal relationship was very probable/confirmed. The causes of death which can be considered vaccine-related were the following: VITT (27 cases with ChAdOx1, 4 cases with Ad26.COV2.S, and 1 case with mRNA1273), myocarditis (two cases with BNT162b2, one case with mRNA1273), acute disseminated encephalomyelitis (ADEM, one case with ChAdOx1), myocardial infarction (one case with Ad26.COV2.S), and complications of rhabdomyolysis (one case with mRNA1273). The ratio of deaths to the total number of administered vaccine doses (55 vs. millions of doses) clearly shows how rare fatal adverse events are, as has been found for other vaccines (for example, quadrivalent live attenuated and trivalent inactivated influenza vaccines) [57,58,59]. Moreover, the effectiveness of the vaccination programs in preventing hospital admission and COVID-19-related deaths has already been demonstrated [60,61]. Vaccines are the principal preventive strategy. Moreover, recent studies have shown the efficacy of new therapies in controlling the disease: monoclonal antibodies and antiviral drugs (e.g., molnupiravir, nirmatrelvir/ritonavir) [62]. In the future, the development of more effective vaccines (e.g., sterilizing immunity), the high percentage of vaccinated people—also in the developing countries—and effective therapeutic protocol will make COVID-19 a disease to live with [63].

A brief discussion about the assessment of the link between the vaccines and the deaths (causal or only temporal) is provided below.

### 4.1. Vaccine-Induced Immune Thrombotic Thrombocytopenia

VITT is characterized by moderate to severe thrombocytopenia associated with venous and/or arterial thrombosis mainly in unusual locations, such as the cerebral venous sinus or the splanchnic veins [64]. The serum of the patients affected by VITT contains autoantibodies against the platelet factor 4 (PF4) antigen that can be identified by PF4 enzyme-linked immunosorbent assay (ELISA) [65]. VITT features resemble those of autoimmune heparin-induced thrombocytopenia (HIT) [25,33,66]. The production of PFA autoantibodies induces platelet activation through the formation of PF4-polyanion complexes, causing thrombocytopenia, disseminated intravascular coagulation, and atypically located thrombotic events. Even if the reason why the vaccine triggers the production of autoantibodies has not been disclosed yet, the causal relationship between VITT and adenoviral vector COVID-19 vaccines is confirmed [67]. Interestingly, we found a case of VITT related to an mRNA vaccine [31]. Even if this case is anecdotal, it suggests that VITT is not only related to adenoviral vectors and that other factors are implicated in its pathophysiology.

To confirm VITT in suspected cases, several recommendations have been published and diagnosis is based on anti-PF4 antibodies testing [68,69]. Therefore, the causal relationship between the death and the vaccine in such cases is quite easy to demonstrate or exclude.

### 4.2. Myocarditis

Myocarditis is a myocardium inflammation with myofiber damage and necrosis [70,71]. It is caused by various events, e.g., viral infections, drugs, etc., and it can lead to severe symptoms and death, even in young people [72,73]. Myocarditis has been reported as a side effect of vaccinations, such as influenza, hepatitis B, etc. [74]. So, it is not surprising that COVID-19 vaccines could also cause myocarditis. We found three cases of death from mRNA vaccine-related myocarditis. Cases of non-fatal myocarditis and pericarditis have also been reported [75,76,77,78,79,80,81]. Indeed, EMA warned about the risk of myocarditis and pericarditis with COVID-19 mRNA vaccines [52]. Moreover, the CDC Advisory Committee on Immunization Practices recognized that COVID-19 mRNA vaccines can cause myocarditis [82]. However, such events are rare, and even COVID-19 itself can cause myocarditis and pericarditis [83,84,85,86]. Therefore, the vaccine’s benefits overcome the risks [87]. When a case of myocarditis or pericarditis occurs after a vaccination and without an alternative cause, a causal link with the vaccine is generally considered to be present or at least possible, even if a specific diagnostic test or laboratory analysis is not available [88,89]. Ante-mortem and post-mortem investigations can demonstrate features of myocarditis (for example, histologically) but cannot differentiate its etiology.

### 4.3. Acute Disseminated Encephalomyelitis

ADEM is an inflammation of the central nervous system associated with demyelination [90,91]. The clinical trials performed to evaluate the safety of the COVID-19 vaccines before general administration did not highlight cases of ADEM [92,93]. However, the AstraZeneca initial trial was provisionally stopped due to two cases of transverse myelitis that eventually were not considered to be undoubtedly related to the vaccine [94]. In the case of death due to ADEM after Vaxzeria administration reported in this review, the pathological mechanism that induced the encephalomyelitis was not clear and the authors did not state with certainty that it was causally related to the vaccine [28]. Another case of neuroinflammatory disorder after a COVID-19 mRNA vaccine (Comirnaty) has been published and some cases of ADEM have been reported in some international and national side effects reporting systems, such as the Vaccine Adverse Event Reporting System (VAERS) and EudraVigilance [95,96]. In such situations, given the fact that only anecdotal cases are reported in the literature, it is not possible to demonstrate a causal link with the vaccine, even in the absence of alternative causes. The risk of ADEM or other severe neurological disorders seems to be extremely low with COVID-19 vaccines. Vice versa, COVID-19 patients can manifest neuropathological features due to the infection, so the balance between risks and benefits is in favour of the vaccine [97].

### 4.4. Rhabdomyolysis

Rhabdomyolysis is due to the damage of the muscle that induces the release of intracellular myoglobin into the blood [98]. The consequent accumulation of myoglobin in the kidneys causes tubular necrosis and acute renal failure [99,100]. In the case of death after a COVID-19 vaccine (mRNA1273) described by Ajmera, the patient was hospitalized because of rhabdomyolysis, developed pneumonia and other complications, and then died [18]. Post-mortem investigations were not performed. Other cases of rhabdomyolysis after COVID-19 vaccination have been reported, both in the literature and in the side effects reporting systems (e.g., VAERS) [101,102,103,104,105,106,107,108,109]. Again, it is difficult to assess whether the link between the rhabdomyolysis is causal or only temporal and the diagnosis of vaccine-induced rhabdomyolysis is based on the exclusion of other causes.

### 4.5. Myocardial Infarction

The commonest cause of acute myocardial infarction is coronary artery occlusion due to the rupture and/or erosion of a vulnerable plaque, with consequent thrombus formation [110,111,112,113,114,115,116]. During the COVID-19 outbreak, there has been a reduction of cases of patients presenting with myocardial infarction [117,118]. However, even some cases of myocardial infarction induced by SARS-CoV-2 infection have been described [119]. The underlying pathological mechanism may involve acute plaque alterations induced by inflammation and cytokines [120,121,122]. Concerning COVID-19 vaccines, we found only one case of death due to acute myocardial infarction possibly related to Janssen vaccine administration [32]. Actually, the case is about a 69-year-old man who developed CVS thrombosis with anti-PF4 autoantibodies alongside coronary thrombosis. Myocardial histology showed fresh myocardial ischemia, while the cerebral tissue did not show any significant alteration. The authors attributed the death to acute myocardial infarction. The man died at home, so clinical information is not available. This is a peculiar case because the patient could also have been diagnosed with VITT, even if it was not the cause of death. The coronary thrombosis may also be related to the pro-coagulation state induced by the antiPF4 autoantibodies. Indeed, antiPF4/heparin antibodies seem to be an independent predictor of myocardial infarction in patients with acute coronary syndrome, suggesting that it may be involved somehow in the pathophysiology of such disease [123].

In any event, we must be clear that in our review we found other cases of myocardial infarction, both acute and recurrent, after COVID-19 vaccines, but in such cases the authors did not state there was a causal link. The relationship was considered to be only temporal. Edler et al. described the case of an old man with several comorbidities who was found dead at home two days after receiving a first dose of Comirnaty^®^ (see Table 1) [23]. The autopsy revealed features of peripheral pulmonary embolism and recurrent myocardial infarction. Schneider and colleagues reported three cases [32]. The first is that of an obese 34-year-old woman who was found dead at home the day after receiving a first dose of Vaxzevria^®^. Fresh myocardial ischemia along with myocardial scars and hypertrophy were the main autoptic findings. The second case regards a 57-year-old man who died two days after Vaxzeria^®^ administration (unknown dose). Again, the autopsy revealed cardiac hypertrophy, myocardial scars, coronary sclerosis, and a fresh myocardial infarction. The third case is that of a 72-year-old woman who died in the vaccination center soon after Comirnaty^®^ administration. The first hypothesis was an anaphylactic reaction, but the autopsy showed coronary sclerosis, myocardial scars, coronary thrombosis, and acute myocardial infarction, while the anaphylaxis diagnostics were negative. In these cases, it was not possible to establish a causal relationship between the vaccine and acute myocardial ischemia, so the causal relationship was not determined.

The relatively high frequency of death and sudden death due to myocardial infarction and the high number of administered COVID-19 vaccine doses each day may suggest that there is only a casual and chronological relationship between the two events. In any case, we think further studies are needed to deepen this topic. We suggest in such cases to perform the VITT diagnostics and to look for anti-PF4 autoantibodies, as in the case previously described, to verify if they could be involved in myocardial infarction after the COVID-19 vaccination.

A limit of our study is the small sample size and the fact that the cases collected in this review do not represent the whole number of deaths after COVID-19 vaccine. We think there could be a sort of “reporting bias” concerning this type of event. At first, researchers could be inhibited from reporting cases of vaccine-related death because of the fear of being held up as “no-vax”. In addition, we noticed that authors tended to be prudent in stating whether or not there was a causal correlation between a vaccine and a death. In fact, in eight cases the causal relationship was not explicitly established. Therefore, post-mortem investigations should be considered essential since the autoptic data and histological analysis could provide more information about the pathological features in such cases of death.

## 5. Conclusions

Given the current pandemic situation, it is fundamental that most people get vaccinated. Concerns about vaccines’ side effects and consequent hesitancy are serious factors that slow down the immunization campaign; thus, the scientific community needs to be cohesive in maintaining that vaccination is fundamental to containing the spread of SARS-CoV-2 [124,125,126,127]. However, it is just as important to evaluate the safety of these vaccines and continuously monitor potential side effects. In this study, we examined 55 cases of death following COVID-19 vaccination reported in the literature to analyze all the available data on fatal cases and assess the existence of a possible correlation with vaccine administration. Given the small number of severe adverse reactions and deaths reported, it is beyond doubt that the benefits of vaccination outweigh the risks. Nevertheless, we think more studies are needed to deepen understanding of possible vaccine-related pathophysiological mechanisms, and researchers should report such cases. Furthermore, we want to encourage post-mortem investigations in cases of death following COVID-19 vaccination because they are essential to better clarify whether a causal relationship between vaccination and death does exist.

## Figures and Tables

**Table 1:** A summary of the main information obtained from the results of our literature review. AAT indicates acute aortic thrombosis; ADEM, acute disseminated encephalomyelitis; AF, atrial fibrillation; CBN, contraction band necrosis; CeVD, cerebrovascular disease; CI, cardiac insufficiency; CND, chronic neurologic disorder; COPD, chronic pulmonary disease; COVID-19, coronavirus disease 2019; CRP, C-reactive protein; CRF, chronic renal failure; CSF, cerebrospinal fluid; CVST, cerebral venous sinus thrombosis; DIC, disseminated intravascular coagulation; DM2, type 2 diabetes mellitus; DVT, deep vein thrombosis; FXIII, coagulation factor XIII; FVL, Factor V Leiden; HL, hyperlipidemia; HT, hypertension; IHD, ischemic heart disease; IL, interleukin; LN, lymph node; MI, myocardial infarction; MTHFR, methylenetetrahydrofolate reductase; NP, not performed; NS, not specified; PAD, peripheral artery disease; PAE, pulmonary embolism; PC, pseudomembranous colitis; PF4, platelet factor 4; PSC, primary sclerosing cholangitis; RA, rheumatoid arthritis; SplVT, splanchnic vein thrombosis; SVT, superficial vein thrombosis; VITT, vaccine-induced immune thrombotic thrombocytopenia. ↓ indicates reduction of levels.

References	N.	Sex	Age (yrs)	Pre-Existing Conditions	Type of Vaccine	Vax–Symptoms Interval	Clinical Manifestations/ Ante-Mortem Findings	Post-Mortem and/or Autoptic Findings	Cause of Death	Causal Relationship?	Hypothetical Pathophysiology
Ajmera 2021 [18]	1	F	85	RA, HL (on statin therapy), asthma, cerebrovascular accident 2 months earlier	Spikevax *II dose*	Soon after (2 days ^╪^)	Rhabdomyolysis complicated by pneumonia during hospitalization	NP	NS	NS	Immune-mediated
Bjørnstad-Tuveng et al. 2021 [19]	1	F	30s	Preeclampsia and huge bleeding during childbirth 11 months before	Vaxzevria *unknown dose*	7 days (10 days ^╪^)	↓ PLTs + intracerebral hemorrhage	CVST (small thrombi) + anti-PF4 antibodies	VITT	Probable	Immune-mediated
Castelli et al. 2021 [20]	1	M	50	Heterozygous MTHFR mutation (C677T)	Vaxzevria *I dose*	7 days (11 days ^╪^)	↓ PLTs, fibrinogen, FXIII + CVST + intracerebral hemorrhage	NP	CVST + intracerebral hemorrhage	NS	NS
Choi et al. 2021 [21]	1	M	22	Elevated blood pressure in two previous measurements	Comirnaty *I dose*	5 days	Chest pain, VF the day after	Atrial myocarditis histological features + non-inflammatory single-cell necrosis + diffuse CBN	Myocarditis	Possible	Immune-mediated (cytokine-mediated or histiocyte-linked immunologic injury)
D’Agostino et al. 2021 [22]	1	F	54	Meniere’s disease	Vaxzevria *unknown dose*	12 days ^╪^	CVST and DIC (arterial and venous)	NP	CVST and DIC	Possible	NS
Edler et al. 2021 [23]	3	F	Elderly *	IHD, CI, HT, dementia, hyperthyroidism, pulmonary emphysema, PC	Comirnaty *I dose*	3 days (5 days ^¥^)	Fever three days after vaccination, then deteriorated and died	Leg DVT + PAE + cerebral infarct + ↑ CRP and IL-6 (consistent with PC)	PAE	No	-
		M	Elderly *	CRF, anemia, AF, PAE, HT, PAD, CeVD, RA, previous prostate carcinoma, chronic pancreatitis	Comirnaty *I dose*	7 days	COVID-19 pneumonia (positive nasopharyngeal swab 12 days after vaccination)	Lung histology consistent with COVID-19 pneumonia	COVID-19		
		M	Elderly *	HT, IHD, DM2, CeVD, dementia, COPD, CRF	Comirnaty *I dose*	2 days ^¥^	Unknown (found dead at home)	Peripheral PAE (mostly organized, some fresh) + swollen axillary LNs (near injection site)	Recurrent MI + IHD		
Franchini et al. 2021 [24]	1	M	50	Heterozygous MTHFR mutation (C677T), folate deficiency	Vaxzevria *I dose*	7 days	↓ PLTs, fibrinogen, FXIII + anti-PF4 antibodies + CVST + intracerebral hemorrhage	NP	CVST + intracerebral hemorrhage	NS	Immune-mediated (autoimmune or protein spike-mediated)
Greinacher et al. 2021 ^§^ [25]	6 ^+^	F	49	None	Vaxzevria *I dose*	5 days	↓ PLTs, fibrinogen + anti-PF4 antibodies + SplVT + peripheral PAE	CVST	VITT	Yes	Immune-mediated (autoantibodies or vaccine-induced antibodies that cross-react with PF4 and PLTs)
		-	-	CND	Vaxzevria *I dose*	7 days	↓ PLTs + anti-PF4 antibodies + CVST	NS	VITT		
		-	-	None	Vaxzevria *I dose*	8 days	↓ PLTs + anti-PF4 antibodies + CVST	Widespread microvascular thrombosis	VITT		
		-	-	None	Vaxzevria *I dose*	16 days	↓ PLTs, fibrinogen + anti-PF4 antibodies + CVST	Multiple organ thrombi	VITT		
		-	-	None	Vaxzevria *I dose*	11 days	↓ PLTs, fibrinogen + anti-PF4 antibodies + CVST + SVT	NS	VITT		
		-	-	Unknown	Vaxzevria *I dose*	12 days	Found dead	Cerebral hemorrhage	VITT		
Jamme et al. 2021 [26]	1	F	69	HT	Vaxzevria *I dose*	11 days	↓ PLTs + anti-PF4 antibodies + CVST + intracerebral hemorrhage + segmentary PAE	NP	CVST + intracerebral hemorrhage	NS	NS
Mehta et al. 2021 [27]	2	M	32	None	Vaxzevria *I dose*	9 days	↓ PLTs, fibrinogen + CVST + intracerebral hemorrhage	NP	CVST + intracerebral hemorrhage	NS	Immune-mediated
		M	25	PSC, migraines, heterozygous FVL mutation (c.1601G>A)	Vaxzevria *I dose*	6 days	↓ PLTs, fibrinogen + anti-PF4 antibodies + CVST + intracerebral hemorrhage	NP	CVST + intracerebral hemorrhage		
Permezel et al. 2021 [28]	1	M	63	DM2, IHD, AF	Vaxzevria *I dose*	12 days ^╪^	ADEM	Diffuse acute demyelination (perivenular) with sparse lymphocytes	ADEM	NS	NS
Pomara et al. 2021 [29]	2	M	50	None	Vaxzevria *I dose*	10 days ^╪^	↓ PLTs, fibrinogen + anti-PF4 antibodies + SplVT + intracerebral hemorrhage	Multi-organ small and medium vessels thrombi + multi-organ endothelial activation	VITT	Yes	NS
		F	37	None	Vaxzevria *I dose*	10 days ^╪^	↓ PLTs, fibrinogen + anti-PF4 antibodies + CVST+ intracerebral hemorrhage	Massive upper limb DVT + feet SVT + multi-organ small and medium vessels thrombi + multi-organ endothelial activation	VITT		
Rodriguez et al. 2021 [30]	1	F	37	None	Janssen	7 days	↓ PLTs, fibrinogen + anti-PF4 antibodies + DVT + CVST + intracerebral hemorrhage	NP	VITT	Yes	Immune-mediated (exaggerated response to the vector)
Sangli et al. 2021 [31]	1	M	65	HT, HP	Spikevax *II dose*	10 days (17 days ^╪^)	↓ PLTs + anti-PF4 antibodies + bilateral lower extremities DVT + acute bilateral PAE + acute gluteal hematoma + CVST + upper extremity DVT + lower extremities compartment syndrome + *S. aureus* sepsis	NP	VITT (complicated by sepsis)	NS	NS
Schneider et al. 2021 [32]	18	M	82	NS	Spikevax *I dose*	1 day ^¥^	Unknown (died at home)	Coronary sclerosis + cardiac hypertrophy + MI scars	Pre-existing cardiac changes	No	-
		F	91	NS	Spikevax *I dose*	1 day ^¥^	Unknown (died at home)	Coronary sclerosis + cardiac hypertrophy + MI scars	Pre-existing cardiac changes		
		F	32	NS	Vaxzevria *I dose*	12 days ^¥^	Unknown (died at home)	Massive cerebral hemorrhage + anti-PF4 antibody (VITT)	Massive cerebral hemorrhage	Very probable	Immune-mediated
		F	34	Obesity	Vaxzevria I dose	1 day ^¥^	Unknown (died at home)	Cardiac hypertrophy + MI scars + fresh MI	Recurrent MI	No	-
		F	48	NS	Vaxzevria *I dose*	10 days ^¥^	Unknown (died at the workplace)	Aortic dissection with rupture	Bleeding aorta		
		M	65	NS	Comirnaty *I dose*	11 h ^¥^	Unknown (died at home)	Myocarditis + coronary sclerosis + cardiac hypertrophy + MI scars	Myocarditis	Possible	Immune-mediated
		M	71	NS	Comirnaty *I dose*	1 day ^¥^	Unknown (died at home)	Cardiac hypertrophy + coronary sclerosis	Pre-existing cardiac changes	No	-
		F	57	NS	Spikevax *II dose*	6 days ^¥^	Unknown (died at home)	Coronary sclerosis + fatty liver + high levels of glucose and lactate (in CSF and aqueous humor)	Hyperglycemic coma		
		M	63	NS	Vaxzevria *I dose*	14 days ^¥^	Unknown (died at home)	Coronary sclerosis + cardiac hypertrophy + MI scars + liver cirrhosis	Pre-existing cardiac changes		
		M	61	NS	Vaxzevria *I dose*	day ^¥^	Unknown (died at home)	Coronary sclerosis + cardiac hypertrophy	Pre-existing cardiac changes		
		M	71	NS	Vaxzevria *unknown dose*	10 days ^¥^	NS	DVT + PAE + coronary sclerosis + cardiac hypertrophy + MI scars (VITT-diagnostics negative)	PAE		
		F	38	NS	Vaxzevria *II dose*	8 days ^¥^	Anaphylactic shock during narcosis induction	CVST + multiple fresh thrombi + cardiac hypertrophy + MI + hypoxic brain changes, anti-PF4 antibodies	Anaphylactic reaction to anesthetics (thrombi formed after the brain damage due to the shock)	Improbable	Immune-mediated
		F	72	NS	Comirnaty *I dose*	12 days ^¥^	Unknown (died at home)	Massive cerebral hemorrhage + coronary sclerosis + cardiac hypertrophy (VITT diagnostics negative)	Massive cerebral hemorrhage	No	-
		F	65	NS	Vaxzevria *I dose*	10 days ^¥^	CVST + cerebral hemorrhages	CVST + cerebral hemorrhages + coronary sclerosis + anti-PF4 antibodies	VITT	Very probable	Immune-mediated
		M	79	NS	Comirnaty *II dose*	6 days ^¥^	Unknown (died at home)	DVT + massive PAE + coronary sclerosis + pericarditis + chronic pulmonary emphysema (VITT diagnostics negative)	PAE	No	-
		M	57	NS	Vaxzevria *unknown dose*	2 days ^¥^	NS	Coronary sclerosis + cardiac hypertrophy + MI scars + fresh MI	Recurrent MI		
		F	72	NS	Comirnaty *II dose*	Soon after	Unknown (died in the vaccination center)	Coronary sclerosis + coronary thrombosis + MI scars + fresh MI (anaphylaxis diagnostics negative)	Fresh MI with coronary thrombosis		
		M	69	NS	Janssen	9 days ^¥^	Unknown (died at home)	CVST (but not significant neuropathologic changes) + coronary sclerosis + coronary thrombosis + cardiac hypertrophy + fresh MI + anti-PF4 antibodies	Fresh MI with coronary thrombosis	Possible	Immune-mediated
Schultz et al. 2021 [33]	3 ^+^	F	37	Pollen allergy, oral contraceptive,	Vaxzevria *I dose*	8 days ^╪^	↓ PLTs + anti-PF4 antibodies + CVST + intracerebral hemorrhage	NP	VITT	Yes	Immune-mediated
		F	42	Pollen allergy, contraceptive vaginal ring	Vaxzevria *I dose*	10 days ^╪^	↓ PLTs, fibrinogen + anti-PF4 antibodies + CVST + intracerebellar hemorrhage	NP	VITT		
		F	54	HT, hormone-replacement therapy	Vaxzevria *I dose*	7 days ^╪^	↓ PLTs, fibrinogen + anti-PF4 antibodies + CVST + intracerebral hemorrhage	NP	VITT		
Scully et al. 2021 [34]	7 ^+^	F	55	NS	Vaxzevria *I dose*	6 days ^╪^	↓ PLTs, fibrinogen + anti-PF4 antibodies + SplVT + AAT + intracerebral hemorrhage	NP	VITT	Possible	Immune-mediated
		F	52	NS	Vaxzevria *I dose*	10 days ^╪^	↓ PLTs, fibrinogen + anti-PF4 antibodies	Multiple organs small vessels thrombosis + CVST+ intracerebral hemorrhage	VITT		
		M	38	NS	Vaxzevria *I dose*	14 days ^╪^	↓ PLTs, fibrinogen + anti-PF4 antibodies + massive PAE	NP	VITT		
		M	25	NS	Vaxzevria *I dose*	9 days ^╪^	↓ PLTs, fibrinogen + anti-PF4 antibodies + CVST	NP	VITT		
		M	54	NS	Vaxzevria *I dose*	10 days ^╪^	↓ PLTs, fibrinogen + SplVT + MI	NP	VITT		
		F	22	NS	Vaxzevria *I dose*	10 days ^╪^	↓ PLTs + anti-PF4 antibodies + CVST + intracerebral hemorrhage	NP	VITT		
		F	32	NS	Vaxzevria *I dose*	12 days ^╪^	↓ PLTs, fibrinogen + anti-PF4 antibodies + CVST	NP	VITT		
See et al. 2021 [35]	3 ^+^	NS	NS	Two were obese, none had risk factors for CVST	Janssen	NS	↓ PLTs + CVST + intracerebral hemorrhage	NS	VITT	Possible	Immune-mediated
Verma et al. 2021 [36]	1 ^+^	M	42	NS	Spikevax *II dose*	14 days ^╪^	Tachycardia + ST-segment elevation + global biventricular dysfunction + left ventricular hypertrophy	Myocardial inflammatory infiltrate (macrophages + T cells + eosinophils + B cells)	Fulminant myocarditis	Possible	NS
Total: 19 articles	55	F:M = 24:23 ^£^	Mean 52.74 (range 22–91) ^£^								

* In this work, subjects’ ages were not given for anonymization. ^+^ Only deceased subjects have been included in the table; the original paper counted more cases, but the other subjects survived. ^§^ In this study, age and sex of only one case (index case) were given. ^¥^ Vaccination–death interval. ^╪^ Vaccination–hospital admission interval. ^£^ The sex was specified in 47/55 cases, the age in 43/55 cases.

**Table 2:** The distribution of causes of death per type of vaccine. The cases in which the causal relationship between the vaccine and the death was not demonstrated or improbable have not been included in this table, so the total number of cases that have been considered is 38. ADEM indicates acute disseminated encephalomyelitis; VITT, vaccine-induced immune thrombotic thrombocytopenia.

Vaccine	Causes of Death	N. Cases (Total = 38)
ChAdOx1 nCoV-19 (Vaxzevria^®^, AstraZeneca)	VITT or uncommonly located thrombosis and/or hemorrhage	27 (71.0%)
	ADEM	1 (2.6%)
BNT162b2 (Comirnaty^®^, BioNTech/Pfizer)	Myocarditis	2 (5.3%)
mRNA1273 (Spikevax^®^, Moderna)	VITT	1 (2.6%)
	Myocarditis	1 (2.6%)
	Rhabdomyolysis (with complications)	1 (2.6%)
Ad26.COV2.S (Janssen^®^, Johnson&Johnson)	VITT	4 (10.5%)
	Myocardial infarction	1 (2.6%)

## Data Availability

Not applicable.
